# Ultraviolet‐induced red fluorescence in androgenetic alopecia—indicating alterations in microbial composition

**DOI:** 10.1111/srt.13777

**Published:** 2024-06-20

**Authors:** Li Zhang, Yebei Hu, Bo Xie, Beilei Zhang, Dongfan Wei, Hongyan Zhang, Yi Chen, Shi Chen, Xiuzu Song

**Affiliations:** ^1^ Department of Dermatology Hangzhou Third Hospital Affiliated to Zhejiang Chinese Medical University Hangzhou China; ^2^ Department of Dermatology Hangzhou Third People's Hospital Hangzhou China; ^3^ Department of Dermatology Affiliated Hangzhou Dermatology Hospital Zhejiang University School of Medicine Hangzhou China; ^4^ Department of Dermatology The Children's Hospital Zhejiang University School of Medicine National Clinical Research Center for Child Health Hangzhou China; ^5^ Department of Clinical Laboratory Hangzhou Third People's Hospital Hangzhou China

**Keywords:** androgenetic alopecia, bacteria, *Cutibacterium acnes*, fluorescence, ultraviolet

## Abstract

**Background:**

Ultraviolet (UV)‐induced fluorescence technology is widely used in dermatology to identify microbial infections. Our clinical observations under an ultraviolet‐induced fluorescent dermatoscope (UVFD) showed red fluorescence on the scalps of androgenetic alopecia (AGA) patients. In this study, based on the hypothesis that microbes are induced to emit red fluorescence under UV light, we aimed to explore the microbial disparities between the AGA fluorescent area (AF group) and AGA non‐fluorescent area (ANF group).

**Methods:**

Scalp swab samples were collected from 36 AGA patients, including both fluorescent and non‐fluorescent areas. The bacterial communities on the scalp were analyzed by 16S rRNA gene sequencing and bioinformatics analysis, as well as through microbial culture methods.

**Results:**

Significant variations were observed in microbial evenness, abundance composition, and functional predictions between fluorescent and non‐fluorescent areas. Sequencing results highlighted significant differences in *Cutibacterium* abundance between these areas (34.06% and 21.36%, respectively; *p* < 0.05). Furthermore, cultured red fluorescent colonies primarily consisted of *Cutibacterium spp., Cutibacterium acnes*, *Staphylococcus epidermidis*, and *Micrococcus spp*.

**Conclusions:**

This is the first study to investigate scalp red fluorescence, highlighting microbial composition variability across different scalp regions. These findings may provide novel insights into the microbiological mechanisms of AGA.

## 1 INTRODUCTION

Androgenetic alopecia (AGA) is the most common form of hair loss, affecting up to 50% of both men and women.^1^ The etiology of AGA is associated with a variety of factors including genetics, environmental influences, hormonal levels, and microecology.^2^, ^3^, ^4^, ^5^ Studies have demonstrated that individuals with AGA have a disrupted scalp microecology compared to those without the disease.^4^, ^5^ Notably, regions experiencing hair loss in AGA patients are more susceptible to the infiltration of inflammatory cells, indicative of localized microinflammatory processes.^6^, ^7^ This imbalance in the microecological environment may trigger inflammatory responses, serving as a “stressor” that exacerbates the disease progression.^8^

In dermatological practices, the use of ultraviolet (UV)‐induced fluorescence is a prevalent technique for aiding in disease diagnosis.^9^, ^10^ The Wood's lamp, for instance, exemplifies this application, emitting UV light onto the skin to induce fluorescence. This technique enhances diagnostic accuracy by correlating the distinct characteristics of the fluorescence with visible signs of skin lesions. UV‐induced fluorescence holds particular diagnostic significance in the identification of microbial infections. For example, when illuminated by a Wood's lamp, pityrosporum folliculitis exhibits a characteristic blue‐white fluorescence at the hair follicles, distinguishing it from bacterial folliculitis.^11^ This fluorescence phenomenon is frequently applied in diagnosing scalp conditions, such as tinea capitis.^12^ However, the application of fluorescence detection has not yet been explored in the case of AGA.

According to our clinical observations, we discerned that under an ultraviolet‐induced fluorescent dermatoscope (UVFD), AGA scalps displayed a distinctive red fluorescence, which contrasted markedly with areas lacking such red fluorescence. Previous studies have reported that microbes emit specific fluorescence when exposed to UV light.^13^ Our research was predicated on the hypothesis that scalp red fluorescence in AGA is produced by microbes. In this study, the origin of red fluorescence was investigated through 16S rRNA gene sequencing and microbial culture methods.

## 2 METHODS

### 2.1 Subjects and study design

A total of 36 patients diagnosed with AGA at Hangzhou Third People's Hospital were included from November 2022 to April 2023. The inclusion criteria were: (i) 18–60 years; (ii) presence of AGA fluorescent (AF) and AGA non‐fluorescent (ANF) areas on the scalp; (iii) absence of systemic diseases and scalp‐associated conditions; (iv) no administration of antibiotics, glucocorticoids, or live bacterial formulations, either through topical or systemic routes, for a period of 3 months; and (v) not being pregnant or lactating. The research protocol was approved by the Medical Ethics Review Committee of Hangzhou Third People's Hospital (Approval No. 2022KA059). All patients signed an informed consent form.

### 2.2 Dermatoscopy examination and scalp microbial sample collection

Clinical photographs were taken for all AGA patients. Their scalps were concurrently examined with a dermatoscope functioned by UV emission. The images obtained under this mode were systematically recorded. Samples were obtained by rubbing the AF and ANF areas of the scalp (about 4 cm^2^) with a sterile cotton swab dipped in 0.9% saline solution. The scalp was rubbed for at least 30 s. Swabs were then placed in sterile tubes and stored at −80°C for DNA extraction.

### 2.3 DNA extraction and 16S rRNA gene sequence analysis

Following the manufacturer's protocol, total microbial DNA was extracted from scalp swab samples through CTAB method. The quality of the extracted DNA was assessed by agarose gel electrophoresis. Quantification was performed by a UV spectrophotometer. Using primers (341F: 5′‐CCTACGGGNGGCWGCAG‐3′; 805R: 5′‐GACTACHVGGGTATCTAATCC‐3′) targeting the V3‐V4 regions of the 16S rRNA gene, polymerase chain reaction (PCR) amplification was performed. A total of 12.5 µL Phusion Hot start flex 2× master mix, 2.5 µL of forward primer, 2.5 µL of reverse primer, and 50 ng of template DNA were used for PCR amplification, and ddH_2_O was added to adjust the total reaction volume to 25 µL. PCR amplification conditions included initial denaturation at 98°C for 30 s; denaturation at 98°C for 10 s, annealing at 54°C for 30 s, and elongation at 72°C for 45 s, repeated for a total of 35 cycles; followed by a final elongation at 72°C for 10 min. PCR products were identified using 2% agarose gel electrophoresis. The PCR products were purified using AMPure XT beads (Beckman Coulter Genomics, Danvers, MA, USA) and quantified using Qubit (Invitrogen, USA). The purified PCR products were assessed using an Agilent 2100 Bioanalyzer (Agilent, USA) and the Library Quantification Kit for Illumina (Kapa Biosciences, Woburn, MA, USA), with qualified library concentrations required to be above 2 nmol/L. After gradient dilution of the qualified sequencing libraries, they were mixed in proportion according to the required sequencing volume, and denatured into single strands with NaOH for sequencing. Sequencing was performed using the NovaSeq 6000 sequencing system for 2 × 250 bp paired‐end reads (supported by LC‐Bio Technology Co., Ltd., Hangzhou, China), with the corresponding reagent being the NovaSeq 6000 SP Reagent Kit (500 cycles).

### 2.4 Bioinformatics and statistical analyses

Paired‐end sequencing reads were assigned to their respective samples using unique barcodes and subsequently truncated to remove the barcode and primer sequences. The reads were merged using FLASH, followed by quality filtration with fqtrim (v0.94) to generate high‐quality clean tags. Chimeric sequences were eliminated using Vsearch software (v2.3.4). Feature tables and sequences were derived post‐dereplication with DADA2. For alpha and beta diversity analyses, sequences were normalized by random selection to ensure uniformity across samples. Taxonomic classification was performed based on the SILVA database (release 138), and the relative abundance of each feature was normalized per sample. Alpha diversity, assessed using Good's Coverage, Chao1 index, observed species, Shannon index, and Simpson index, was calculated with QIIME2 to evaluate the species diversity complexity within each sample. Beta diversity was plotted by principal coordinates analysis (PCoA) based on weighted UniFrac distances and calculated by QIIME2, the graphs were drawn by R package. Sequence alignment was performed using Blast, with representative sequences annotated via the SILVA database. Additional data visualizations were generated using R package version 3.5.2. Based on the obtained species abundance statistical information, differential analyses between comparison groups were conducted. Specifically, for comparisons between two groups of samples with biological replicates, the Mann–Whitney U test was employed. Functional prediction of bacterial communities was performed using PICRUSt2, which utilizes KEGG databases to infer potential metabolic pathways. The statistical significance of pathway variability between sample groups was analyzed using STAMP, employing a *t*‐test. The threshold for significance in all statistical analyses was set at *p* < 0.05 (*p < 0.05, **p < 0.01, ***p < 0.001, ****p < 0.0001).

### 2.5 Microbial culture and detection

Swabs collected from the AF areas of 5 AGA patients were uniformly spread on Columbia blood agar plates for both aerobic and anaerobic culturing. Bacterial colonies grown under these conditions were observed under a Wood's lamp. Colonies emitting red fluorescence were isolated under the same culturing conditions for further cultivation. Cultures from individual bacterial colonies on various agar plates were subjected to Matrix‐assisted Laser Desorption/Ionization Time‐of‐flight Mass Spectrometry (MALDI‐TOF MS) analysis using the BRUKER MALDI‐TOF MIRCROFLEX LT/SH (Bruker Corporation, Germany) for species identification. Special attention was given to colonies emitting red fluorescence, which were subjected to Gram staining and microscopic examination. The acquired raw mass spectra were cross‐referenced with the IVD Library to discern the bacterial species within the samples. A confidence score ≥1.7 was considered relatively reliable. In the identified red‐fluorescent colonies presented below, all had confidence scores ≥1.7.

## 3 RESULTS

### 3.1 Clinical observation of patients

**FIGURE 1:**
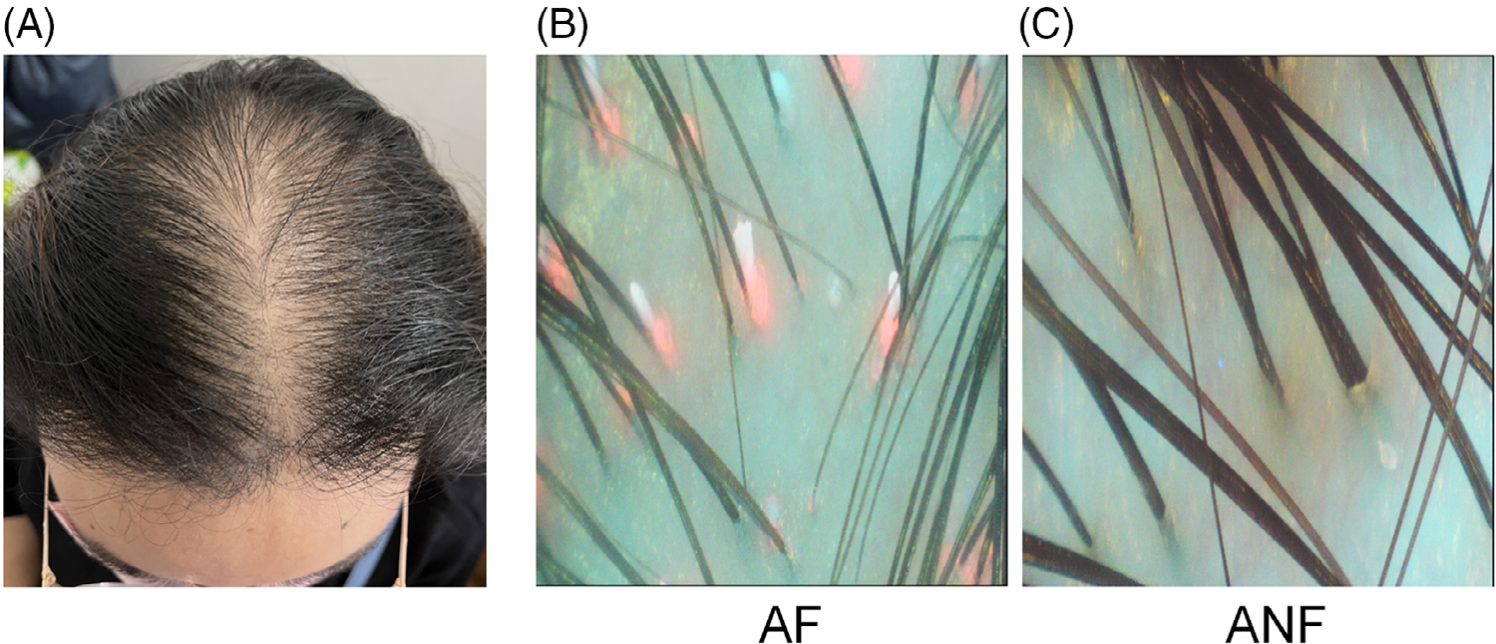
Clinical photograph and dermoscopic images with ultraviolet emission of patients with androgenetic alopecia (AGA). A clinical photograph of a patient meeting the inclusion criteria (A), along with dermoscopic observations of AGA fluorescent area (AF, B), and AGA non‐fluorescent area (ANF, C).

Observation of AGA (Figure 1A) with a UVFD revealed distinct areas of fluorescence and non‐fluorescence (Figure 1B,C). Significant red fluorescence was observed in the fluorescent areas, while the non‐fluorescent scalp regions displayed no fluorescence, creating a stark contrast.

### 3.2 Scalp microbial diversity

**FIGURE 2:**
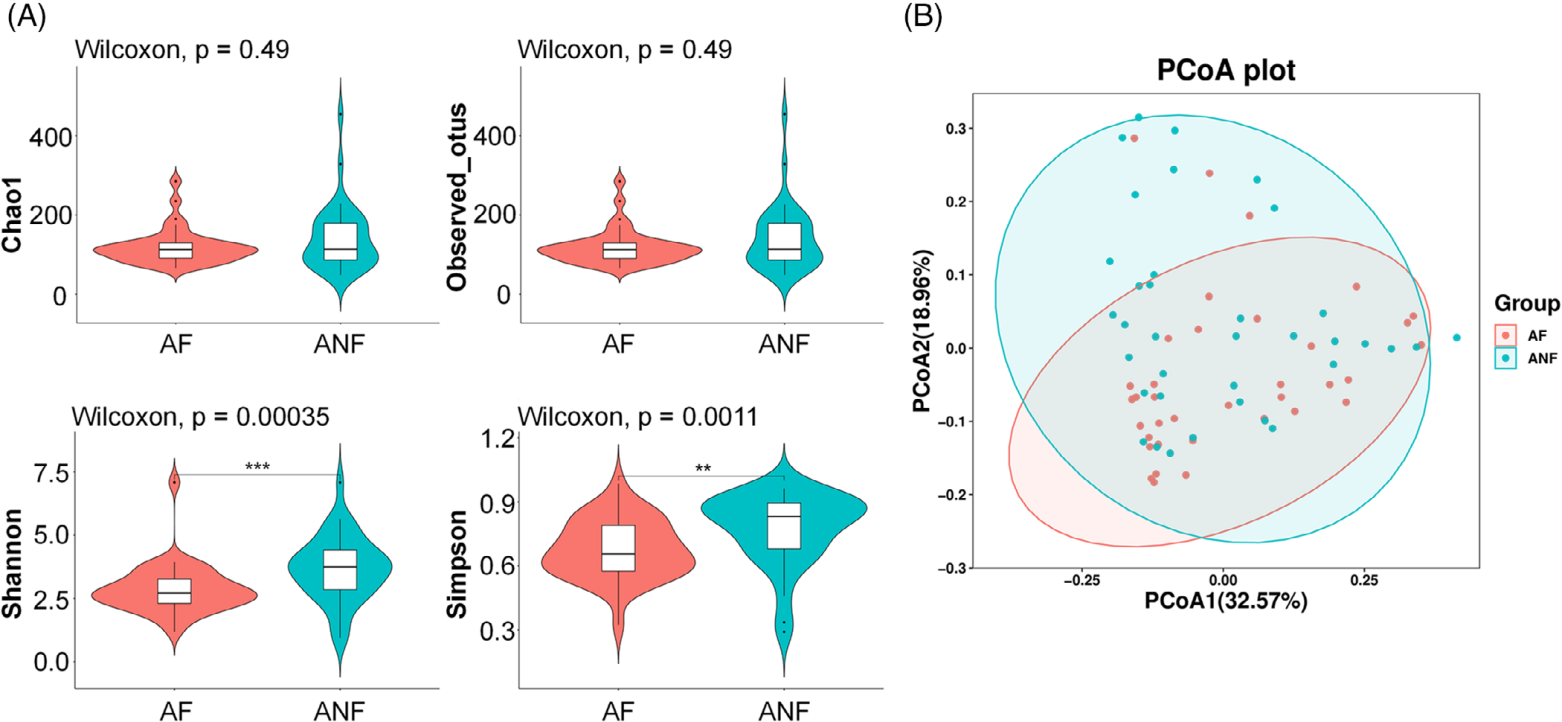
Alpha diversity and beta diversity of bacteria in androgenetic alopecia (AGA) fluorescent area (AF) and AGA non‐fluorescent area (ANF). Alpha diversity (A) reflects species richness and evenness in a given environment and includes Chao1 index, observed species, Shannon index, and Simpson index. Principal coordinates analysis (PCoA, B) based on weighted UniFrac distance reflects intergroup heterogeneity, and the different colored points in this PCoA plot represent samples from different groups. Every point symbolizes an individual skin microbiome sample. The significance of differences in alpha diversity was assessed using the Mann–Whitney U test (*p < 0.05, **p < 0.01, ***p < 0.001).

We assessed the alpha diversity of our samples using Good's Coverage, Chao1 index, observed species, Shannon index, and Simpson index (Figure 2A). Good's Coverage indicated the representativeness of our sequencing results to the actual sample composition, and our findings demonstrated a high sequencing coverage (>99%). Utilizing the Chao1 index and observed species, which are primarily employed to estimate the number of species within a community, the analysis revealed that there were no significant differences in species numbers between the AF and ANF groups (all *p >* 0.05). The Shannon index and Simpson index, which consider both species richness and evenness, revealed that the AF group was significantly lower than the ANF groups (all *p *< 0.05). This suggested that despite the similar number of species between the two groups, the evenness in the AF group was lower.

Beta diversity reflected intergroup heterogeneity. Given that all samples were collected from the scalp environment, we utilized weighted UniFrac distance matrix to assess the beta diversity of the samples. PCoA results demonstrated corresponding overlaps and separations among the two groups (Figure 2B).

### 3.3 Scalp bacterial community composition

**FIGURE 3:**
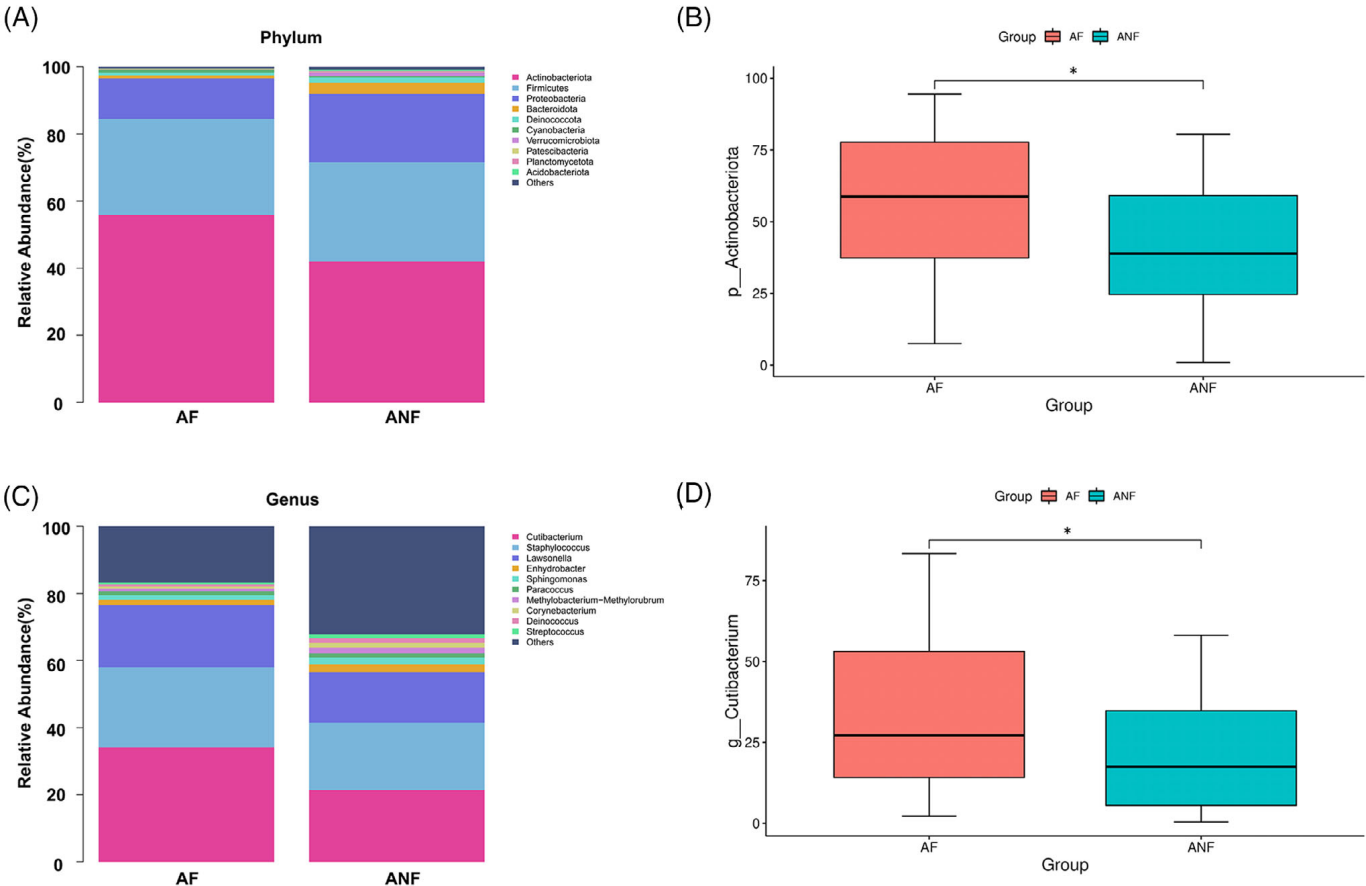
Combined analysis of microbial composition and the relative abundance of individual bacterial taxa in androgenetic alopecia (AGA) fluorescent area (AF) and AGA non‐fluorescent area (ANF). The bar stacked graphs show the top 10 species composition at the phylum (A) and genus (C) levels for two groups. Further detailed analysis reveals the specific findings for key bacterial taxa. The relative abundance of Actinobacteriota (B) and *Cutibacterium* (D) in the AF and ANF groups showcases these differences. For comparisons between the two groups, the Mann–Whitney U test was used (*p < 0.05).

We analyzed the bacterial composition of two sample groups at various levels. The following focused primarily on the phylum and genus levels (Figure 3). At the phylum level, Actinobacteriota, Firmicutes, and Proteobacteria were the most common, collectively accounting for over 90% of the total composition (Figure 3A). The phylum‐level significant difference analysis (Figure 3B) revealed that the relative abundance of Actinobacteriota in the AF group was significantly higher than in the ANF group. Conversely, the relative abundance of Proteobacteria was higher in the ANF group than in the AF group. Other phyla with statistically significant differences were present in smaller relative abundances.

At the genus level, *Cutibacterium* and *Staphylococcus* were the predominant genera in the AF and ANF groups. In the AF group, their proportions were 34.06% and 23.99%, respectively, whereas in the ANF group, they showed comparable abundances of 21.36% and 20.14% (Figure 3C). The genus‐level significant difference analysis (Figure 3D) revealed a notable change in the relative abundance of *Cutibacterium* across the two groups. The abundance of other differentially abundant genera was lower.

### 3.4 Functional prediction of microbiota

**FIGURE 4:**
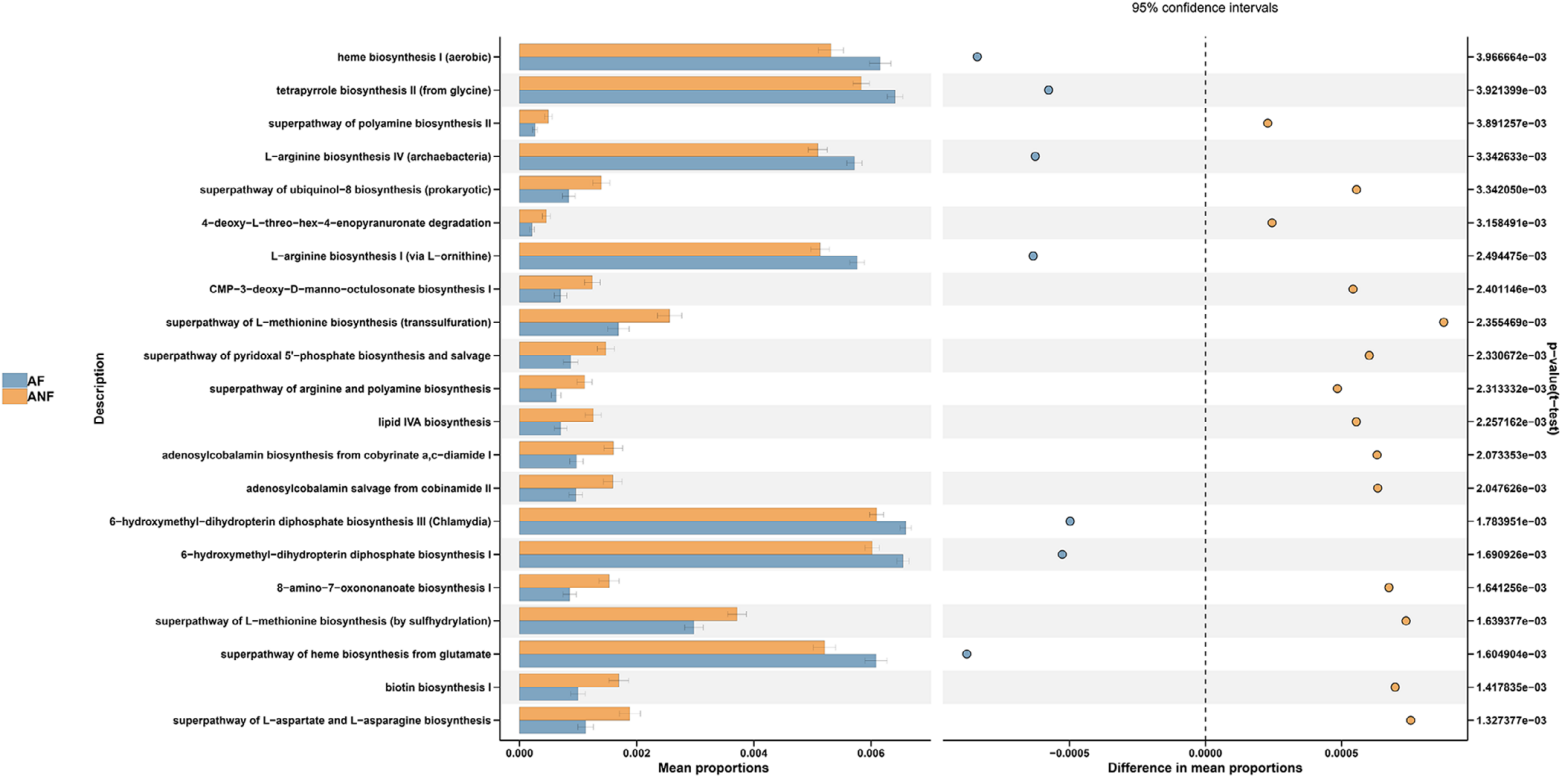
Evaluation of distinct bacterial groups using KEGG Pathway analysis to identify pathways that exhibit significant differences between the androgenetic alopecia (AGA) fluorescent area (AF) and AGA non‐fluorescent area (ANF). Top items are presented, complete with their appropriate 95% confidence intervals and *p*‐values.

To assess the functional shifts within the scalp microbiome, the PICRUSt2 algorithm was employed to interpret 16S rRNA gene sequence information, facilitating the prediction of metabolic activities (Figure 4). In comparison to the ANF group, the AF group demonstrated a pronounced enrichment of several metabolic pathways, including superpathway of heme biosynthesis from glutamate, 6‐hydroxymethyl‐dihydropterin diphosphate biosynthesis, tetrapyrrole biosynthesis II (from glycine), and heme biosynthesis I (aerobic). In contrast, the ANF group exhibited a greater enrichment in pathways, including but not limited to superpathway of L‐aspartate and L‐asparagine biosynthesis, biotin biosynthesis I, and superpathway of L‐methionine biosynthesis (by sulfhydrylation).

### 3.5 Cultivation of scalp swab bacteria and red fluorescent colonies observed under Wood's lamp

**FIGURE 5:**
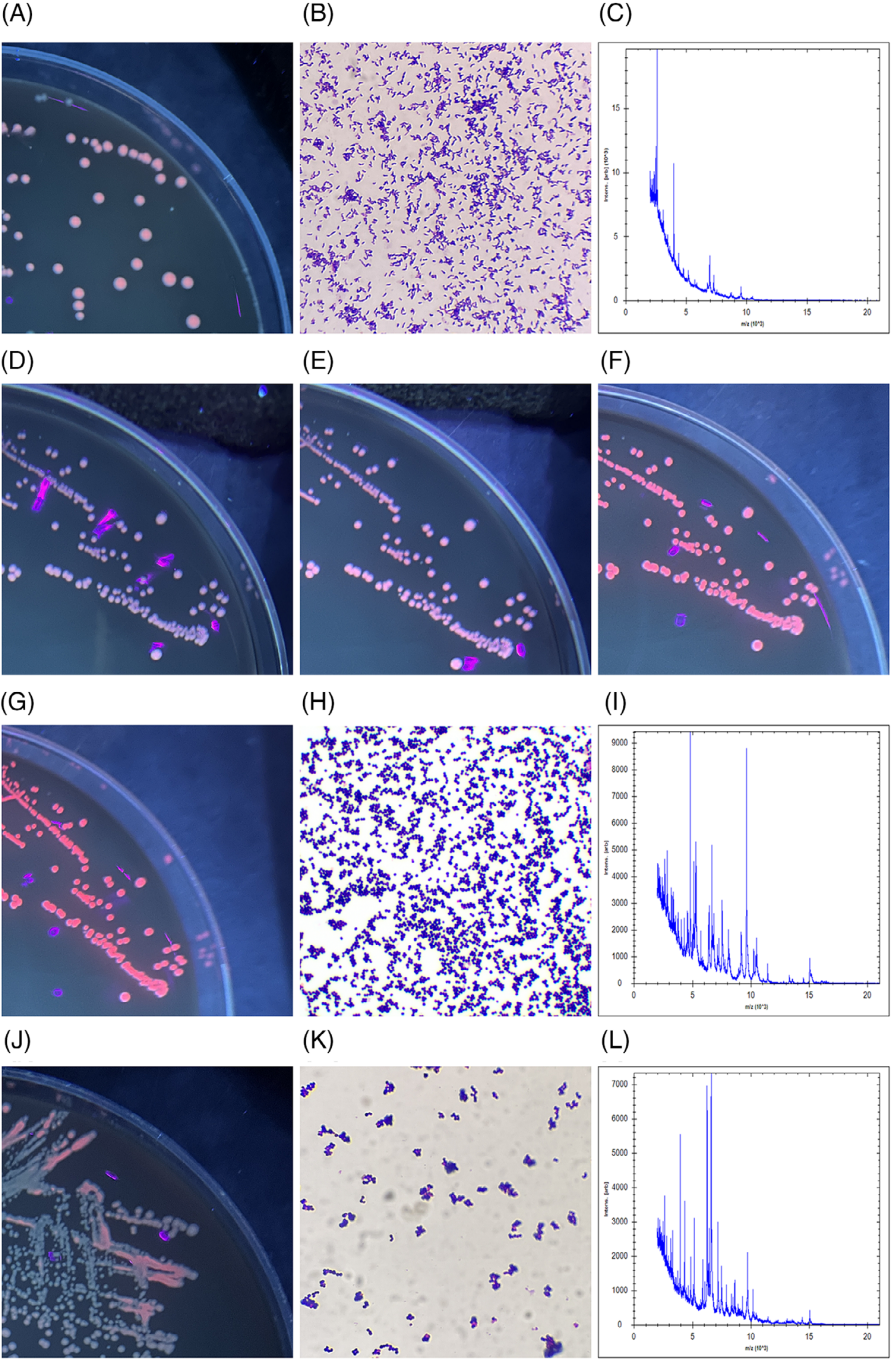
Colonies that exhibited red fluorescence under a Wood's lamp were analyzed, revealing their characteristics through Gram staining and mass spectrometry identification. Under anaerobic conditions, three types of red‐fluorescent colonies were detected. The first type was identified as *Cutibacterium spp*. (A–C), as evidenced by its Gram staining results (B) and validated through mass spectrometry (C). The second type was specifically confirmed as *Cutibacterium acnes*, exhibiting characteristic Gram‐positive properties. (Note: Due to the overlap of red fluorescent colony images between *Cutibacterium spp*. and *Cutibacterium acnes*, and the superior clarity of the former, additional images of *Cutibacterium acnes* are not included to avoid redundancy). The third type, *Staphylococcus epidermidis* (D–I), demonstrated a notable enhancement in red fluorescence when exposed to air. This change was documented sequentially: immediately after air exposure (D), then at 30 s (E), 1 min (F), and finally at 2 min (G). This was complemented by Gram staining of the colony (H) and confirmed through mass spectrometry (I). For the aerobically cultured colonies, those displaying red fluorescence were identified as *Micrococcus spp*. (J–L), with this identification being substantiated by Gram staining (K) and further confirmed through mass spectrometry (L). (Note: The purple spots in the culture image are reflections of the Wood's lamp bulbs).

Cultured colonies exhibiting red fluorescence were scrutinized by a Wood's lamp. These distinct red fluorescent colonies were isolated and cultivated under uniform conditions (either anaerobic or aerobic) until the formation of pure colonies, which were then re‐examined under Wood's lamp (Figure 5A,D‐G,J). Under anaerobic conditions, three varieties of red fluorescent colonies were discerned. The first variety was identified as *Cutibacterium spp*. (Figure 5A–C), characterized as Gram‐positive bacteria (Figure 5B). The second variety was specifically identified as *Cutibacterium acnes* (*C. acnes*), also Gram‐positive (Note: due to the overlap of red fluorescent colony images between *Cutibacterium spp*. and *C. acnes*, and the superior clarity of the former, additional images of *C. acnes* are not included to avoid redundancy). The third variety was identified as *Staphylococcus epidermidis* (*S. epidermidis*) (Figure 5D–I), which was also Gram‐positive (Figure 5H). Interestingly, *S. epidermidis* cultured anaerobically initially exhibited weak red fluorescence, which significantly intensified within minutes upon air exposure. The images illustrated a progressive intensification of the red fluorescence in *S. epidermidis*, commencing immediately upon exposure to air (Figure 5D), then observed at 30‐s intervals (Figure 5E), at 1 min (Figure 5F), and finally at 2 min (Figure 5G). This result aligned with the findings of Xu et al.^13^ Aerobically cultured red fluorescent colonies were identified as *Micrococcus spp*. (Figure 5J–L) and were Gram‐positive (Figure 5K).

## 4 DISCUSSION

UV light‐induced fluorescence, along with other fluorescence‐based techniques, is instrumental in dermatology for diagnosis and treatment.^14^ In our study, scalp swab analysis revealed a significantly higher presence of *Cutibacterium* in the AF group compared to the ANF group. Microbial cultures emitting red fluorescence included *Cutibacterium spp*., *C. acnes*, *S. epidermidis*, and *Micrococcus spp*., aligning with previous research findings.^13^ However, we noted inconsistencies between culture and sequencing results, possibly due to: (i) Differences in culturing media and scalp microecology, where 16S rRNA gene sequencing identified hard‐to‐culture microbes, offering a truer representation of microbial composition. (ii) *S. epidermidis* fluoresced post‐anaerobic cultivation upon exposure to aerobic conditions, a situation not mimicked in clinical UVFD observations. (iii) *Micrococcus spp*. were present in both the AF and ANF groups; however, there was no statistically significant difference between them, warranting further exploration of its actual state on the scalp. The discrepancies in observed fluorescence between cultures and scalp colonies may stem from environmental factors like sebum, other bacteria, antimicrobial peptides, metabolites, and humidity.^15^ To our knowledge, this is the first study investigating scalp red fluorescence in AGA.

Our findings suggest a strong correlation between scalp red fluorescence and bacteria, primarily attributed to *Cutibacterium species* (further identified as *C. acnes* through mass spectrometry). Previous studies have found that *C. acnes* could induce inflammation. For example, co‐culturing *C. acnes* fractions with human keratinocytes led to an early upregulation of TLR‐2 and TLR‐4, accompanied by increased expression and secretion of MMP‐9 by keratinocytes.^16^ Furthermore, *C. acnes* could activate the type I interferon (IFN‐I) signaling axis in human macrophages by initiating the cGAS‐STING pathway.^17^ Previous reports have associated facial red fluorescence under UV light to coproporphyrin III (CPPIII) and protoporphyrin IX (PPIX) produced by *C. acnes*.^18^, ^19^ Our functional prediction analysis revealed that the tetrapyrrole biosynthesis pathway was more enriched in the microbial community of the AF group. This pathway plays a critical role in the synthesis of porphyrin compounds, constituting the fundamental process for constructing the porphyrin backbone.^20^, ^21^ The biosynthesis of tetrapyrrole and the production of porphyrins also lay the groundwork for the biological synthesis of heme,^20^, ^21^, ^22^ potentially leading to the enrichment of the heme biosynthesis pathway in the AF group. Therefore, it is reasonable to speculate that porphyrins serve as the biochemical foundation for the red fluorescence observed on the scalp of AGA patients. Porphyrins may also induce inflammation.^23^, ^24^, ^25^ Evidence showed that coproporphyrin III exposure led to a rise in interleukin‐8 (IL‐8) expression in human keratinocytes within 3 h, potentially triggering inflammation.^23^ Therefore, scalp fluorescence might serve as an indicator of scalp inflammatory status.

This potential usage of scalp fluorescence in indicating inflammation status leads us to further explore its specific role in the context of AGA. Current research highlights inflammation's prevalence around miniaturized hair follicles in AGA, often more pronounced in severely miniaturized follicles and the isthmic region.^26^ A separate study revealed chronic inflammation in half of AGA cases, characterized by lymphocytes, histiocytes, and occasional plasma cells, with about 40% of samples showing an increased mast cell count. Such inflammation, localized around hair capillaries and accessory structures,^6^ might disrupt follicular function and affect hair growth. Contrary to control samples, which exhibited normal hair growth without follicular inflammation, the transitional areas of hair loss in AGA showed significant immune reactions. These reactions were particularly marked by the infiltration of activated T cells in the lower part of the hair follicle infundibulum and the presence of inflammatory cells in the bulge area.^7^ Such immune activity, especially in regions critical for hair cycle regulation and stem cell location, might be a key factor that disrupts normal hair growth in AGA. Despite varying reports on the specific sites of inflammation, its presence in any part of the hair follicle could impact normal growth. If there is a correlation between inflammation and microbiome changes in the scalp, this might be detectable through the observation of red fluorescence.

In our research, we observed intriguing phenomena. Despite mass spectrometry confirming *C. acnes* identity, some colonies fluoresced under Wood's lamp while others did not during cultivation. The variation might result from differences in subspecies and phylogeny of *C. acnes*. Type I strains produced more porphyrins than types II and III, with the latter rarely fluorescing under Wood's lamp.^24^ Indeed, different *C. acnes* subspecies coexisted on the skin,^27^, ^28^ influencing porphyrin production. Our mass spectrometry could not distinguish these subspecies, indicating a need for further research. We also noted changes in fluorescence over time, likely due to variations in bacterial metabolism.

However, there are limitations to this study. The current literature on facial and hair follicle red fluorescence is inconsistent, with some theories suggesting a connection to sebum secretion.^29^, ^30^ Our study points out that microbes might be responsible for this fluorescence, but does not explore its relationship with sebum production. Moreover, the homogeneity in racial backgrounds and living environments of the participants may limit the generalizability of the findings, considering the influence of these factors on microbial compositions.^31^ It is possible that this does not completely reflect the diversity of the entire human population.

The use of Wood's lamp and UVFD highlights the importance of UV‐induced fluorescence in diagnosing and treating skin diseases.^32^ Hence, understanding the origin of the red fluorescence observed on the scalp under UV light is crucial. Our research suggests that this red fluorescence on the scalp is associated with microbes. 16S rRNA gene sequencing suggests *Cutibacterium* as a potential source, while culturing results link *Cutibacterium spp*., *C. acnes*, *S. epidermidis*, *Micrococcus spp*., to the red fluorescence. Identifying which microbial metabolites cause this phenomenon and the specific wavelengths of red fluorescence they emit are questions we could explore further. For the scalp, what does this red fluorescence signify? Can this phenomenon assist in the diagnosis and treatment of future scalp issues? These are questions worth pondering. In summary, scalp microbiota are intimately associated with scalp health. The observed red fluorescence should not be overlooked, as continued exploration of this phenomenon will undoubtedly enhance our understanding of scalp health.

## CONFLICT OF INTEREST STATEMENT

The authors declare no conflicts of interest.

## ETHICAL APPROVAL

The research protocol was approved by the Medical Ethics Review Committee of Hangzhou Third People's Hospital (Approval No. 2022KA059).

## Data Availability

The data that support the findings of this study are available from the corresponding author upon reasonable request.
